# RETRACTION: Tristetraprolin Attenuates Brain Edema in a Rat Model of Cerebral Hemorrhage

**DOI:** 10.1002/brb3.71871

**Published:** 2026-10-29

**RETRACTION**: P. Li, J. Zhang, X. Li, and H. Gao, “Tristetraprolin Attenuates Brain Edema in a Rat Model of Cerebral Hemorrhage,” *Brain and Behavior* 9, no. 3 (2019): e01187, https://doi.org/10.1002/brb3.1187.

The above article, published online on 06 February 2019 in Wiley Online Library (wileyonlinelibrary.com), has been retracted by agreement between the journal Editor‐in‐Chief, Amanda Bischoff‐Grethe; and Wiley Periodicals, LLC. The retraction has been agreed upon following concerns raised by a third party. An investigation showed that three images presented in Figure 1a, Group I (24 h), Group II (24 h), and Group III (24 h), appear to have been duplicated from a previous article published by a different group of authors. Furthermore, an apparent overlap was found in Figure 3a between the Group II (24 h) and Group IV (24 h) images. The authors were invited to comment and provide their original data; however, no response was received. The editor considers the results and conclusions to be unreliable, and therefore the article must be retracted. The authors did not respond to the retraction notice.

